# Optimization of a syngeneic murine model of bone metastasis

**DOI:** 10.1016/j.jbo.2020.100298

**Published:** 2020-05-31

**Authors:** Henry P. Farhoodi, Aude I. Segaliny, Zachary W. Wagoner, Jason L. Cheng, Linan Liu, Weian Zhao

**Affiliations:** a Sue and Bill Gross Stem Cell Research Center, University of California, Irvine, Irvine, CA 92697, USA; b Department of Pharmaceutical Sciences, University of California, Irvine, Irvine, CA 92697, USA; c Chao Family Comprehensive Cancer Center, University of California, Irvine, Irvine, CA 92697, USA; d Edwards Life Sciences Center for Advanced Cardiovascular Technology, University of California, Irvine, Irvine, CA 92697, USA; e Department of Biomedical Engineering, University of California, Irvine, Irvine, CA 92697, USA; f Department of Biological Chemistry, University of California, Irvine, Irvine, CA 92697, USA

**Keywords:** Cancer, Animal models, Bone metastasis, Intra-arterial, caudal artery, 4T1

## Abstract

Many cancers metastasize to the bones, particularly in cases of breast and prostate cancers. Due to the “vicious cycle” of cancer cells inducing bone resorption, which promotes further tumor growth, they are difficult to treat and may lead to extreme pain. These factors increase the urgency for emerging therapeutics that target bone metastases more specifically and effectively. Animal studies are essential to the development of any therapeutics, but also require robust animal models of human diseases. Robust animal models are often challenging to develop in the case of bone metastasis studies. Previous methods to induce bone metastasis include intracardiac, intravenous, subcutaneous via mammary fat pad, and intraosseous cancer cell injections, but these methods all have limitations. By contrast, the caudal artery route of injection offers more robust bone metastasis, while also resulting in a lower rate of vital organ metastases than that of other routes of tumor implantation. A syngeneic animal model of bone metastasis is necessary in many cancer studies, because it allows the use of immunocompetent animals, which more accurately mimic cancer development observed in immunocompetent humans. Here we present a detailed method to generate robust and easily monitored 4T1-CLL1 syngeneic bone metastases with over 95% occurrence in BALB/c mice, within two weeks. This method can potentially increase consistency between animals in bone cancer metastasis studies and reduce the number of animals needed for studying bone metastases in mice.

## Highlights

• A method to generate bone metastases in over 95% of mice.
• Tumors can be detected within one to two weeks.
• Low rates of vital organ metastases, relative to other methods.
• Consistent tumor localization in lower body.
• Growth rate and consistency of tumors can be controlled by quantity of cancer cells injected.

## 1 Introduction

Over 90% of cancer mortalities can be attributed to metastasis, the dissemination of cancer cells from the primary tumor site to other tissues in the body [1], [2]. Bone metastases are frequent, occurring in up to 70% of patients with advanced breast or prostate cancers and in approximately 15 to 30% of patients with cancers of the lung, colon, stomach, bladder, uterus, rectum, thyroid, or kidney [3], [4]. A consistent and efficient animal model is necessary to study the mechanisms of bone cancer metastasis and develop novel treatments for these bone metastases. Unfortunately, the current standard model, using intracardiac injection of cancer cells, is not easy to perform, nor does it produce metastases specific to bone [5], [6]. Other implantation routes for establishing bone metastasis are not optimal for varying reasons. Intravenous injections (IV) tend to produce lung tumors that can metastasize to bones, but also commonly metastasize to the liver, spleen, or brain. Another pitfall of IV injections is that the relatively large lung tumors, that inevitably develop, can mask weaker signals located in other parts of the body, due to signal detector saturation. This issue is exaggerated for subcutaneous injections to the mammary fat pads (subsequently referred to as just “fat pads”) due to the large primary tumors which form before metastasis, and the fact that the fat pads are in close proximity to the bones of the leg, pelvis, and spine. Fat pad injections also rely on spontaneous dissemination of cancer cells which results in low rates of metastasis to bones, increasing the number of animals needed for experiments, and makes it difficult to establish consistent timelines in experiments. Intraosseous (also known as intratibial) injections are consistent and well controlled in terms of cell quantity/growth but require an invasive bone drilling procedure that creates local inflammation and fails to accurately mimic the natural cancer bone metastatic process from the circulatory system [7], [8]. The methods mentioned above result in tumors formed at the vital organs, causing high animal mortality rates, which impede the study and development of targeted treatments for bone metastasis in animal models [9].

Due to ethical and financial constraints, the number of animals used in an experiment needs to be limited, and an efficient rate of bone metastasis decreases the number of animals needed to complete a study [10], [11]. In recent work, we required a robust syngeneic cancer model to evaluate our cell therapy for treating bone metastasis and the resulting damage caused to bones [12]. We tested each of the previously mentioned models and were unsatisfied with their rates of bone specific metastasis despite using *in vivo* selection to improve bone homing (Table 1).

**Table 1:** List of materials used in this syngeneic model of bone metastasis.

**Materials**	Company	Catalog/Model #
4T1 Murine breast cancer cells	ATCC	CRL-2539
4T1-CLL1 RFP/Luciferase breast cancer cells	N/A	Zhao lab CLL1
Viral vector (RFP/Lucifase lentiviral particles)	GenTarget Inc.	LVP324 CMV-Luciferase (firefly)-2A-RFP (Puro)
Ketamine (KetaVed® C III)	Patterson Veterinary	07-890-8598
Xylazine hydrochloride (Vetranal™)	Sigma Aldrich	46995-100MG
Cotton non-woven gauze pads	Fisher Scientific	22028556
Puromycin	Invivogen	NC9138068
RPMI 1640 Media	Gibco	11875119
Fetal Bovine Serum	Seradigm	1500–500
Penicillin/Streptomycin	Genclone	25–512
Dulbecco’s Phosphate-Buffered Saline	Gibco	14190250
Trypsin (0.25%)	Gibco	25200–056
Trypan Blue	Invitrogen	T10282
Cell Strainers (70 μm)	Falcon	352350
50 mL Conical tubes	Nunc	339652
15 mL Conical tubes	Nunc	339650
Eppendorf Tubes (1.7 mL)	Axygen	14-222-168
29G½ Inch Diabetic syringes (3/10 cc)	ADW Diabetes	SY8881600145
Numbered ear tags	Fisher Scientific	NC0800034
D luciferin potassium salt	Perkin Elmer	122799
27G½ Inch 0.4 × 12 mm hypodermic needles	Air-tite	BD305109
Polypropylene 1 mL Norm-ject luer slip syringes	Henke Sass Wolf	4010.200 V0
Matrigel™	Corning	CB40234A
Black masking tape	Grainger	48UV67
Eclipse Black™ Cardstock 8.5″ x 11″	Astrobrights	45UV16
Isoflurane	Piramal	66794-017-25
**Animals**	Company	Catalog #
Female BALB/cJ mice aged 5–8 weeks	Jackson Laboratories	000,651
**Equipment**	Company	Catalog #
IVIS Lumina Animal Imager	Xenogen	N/A
Hemocytometer	Hausser Scientific	02–671-51B
Mortar and pestle	Neta Scientific	USS-JMD050
Heating mat	Gaymar Industries Inc.	TP650
Fluorescent microscope	Nikon	Eclipse Ti
Mouse restrainer	VWR	10718-054

Intra-arterial injections are sometimes used to generate bone metastases in mice, as described by Wright et al. [9]. This method was recently expanded upon by Kuchimaru et al. to improve bone metastasis in rodents using the caudal artery of the tail. This injection route produces consistent and efficient distribution of cancer cells localized in the leg bones, via the blood distributed to the lower body. Caudal-artery injections are easier to perform than tail vein injections, a common technique used for *in vivo* studies, and result in few complications. In addition, the frequency of metastases to the vital organs is remarkably low, with most tumors establishing themselves in the bone, allowing the majority of mice to survive to the experimental endpoint [13].

Most bone metastasis studies use human cancer lines xenotransplanted to animal models, and thus require immunocompromised animals for the human cancer cells to produce detectable tumors [14]. This immunocompromised system does not accurately mimic the conditions of cancer development and treatment in humans. A syngeneic cancer model enables the study of cancer treatments in an immunocompetent system and can give additional information about the efficacy of an anticancer therapy not possible in immuno-compromised models alone [8]. The 4T1 cell line (ATCC® CRL-2539™) is a murine breast cancer that produces metastatic growth equivalent to human breast cancer metastasis, when given to BALB/c mice [15]. While these cells are excellent for mimicking general metastasis, the rates of bone specific metastasis can be improved by *in vivo* selection for cells that prefer to metastasize to bones [16], [17], [18], [19], [20], [21]. *In vivo* selection was originally developed by the Clézardin lab to generate far higher rates of bone metastasis, using a fluorescence-based reporter system [20]. The use of *in vivo* selection for bone homing cells can be further enhanced by engineering cells to express luciferase. This enables the concurrent selection of cells that luminesce more stably and brightly *in vivo*, grow faster within the bone, and produce a more identifiable bone tumor from which to select cells [21].

Although the caudal artery method (Kuchimaru et al.) is an outstanding effort to improve bone metastatic rates, the previously described method has a broad scope using many cell lines (mostly xenogeneic), and it was necessary to optimize specifics of the protocol for syngeneic experiments [13]. Here, we describe details and comments to establishing robust and consistent 4T1 breast cancer bone metastasis in a syngeneic BALB/cJ mouse model.

## 2 Materials

### 2.1 Methods

Note on animal studies: All experiments involving live animals should be performed under the guidance of an Institutional Animal Care and Use Committee and follow national and local regulations. Experiments in this study were performed at the University of California Irvine under IACUC protocol number AUP-18-134.

### 2.2 *In vivo* bone metastatic cell selection

4T1 cells were transduced to express RFP and luciferase using a multiplicity of infection of 10 (see manufacturer protocol). Engineered 4T1 cells were then mixed with Matrigel™ at a concentration of 1 million cells/mL. 6-week-old female BALB/cJ mouse were fully sedated and their abdomens were shaved to expose mammary fat pads (Supplemental Figure 1a). The cell/Matrigel™ mixture was subcutaneously injected to one or both lower inguinal mammary fat pads at 100,000 cells per mouse. Mice were monitored bi-weekly using *in vivo* bioluminescent imaging. After detecting leg bone metastasis in a mouse, the mouse was sacrificed, and the identified leg bone harvested. *Ex vivo* bioluminescent imaging was performed on the harvested leg to confirm that the metastasis is within the bone (Supplemental Figure 2). Leg bones were cleaned with 70% ethanol and ground to ∼ 1 mm pieces using a mortar and pestle. Growth media (RPMI 1640, 10% FBS, 1% pen/strep) was used to rinse cells and collect them, before being run through a 70 μm cell strainer into a clean 50 mL tube. The filtered cell solution was transferred to a T25 flask and allowed to grow in an incubator (37 °C, 5% CO^2^) overnight. Growth media was changed the following day to selection media (RPMI 1640 containing 10% FBS, 1% pen/strep, and 3 μg/mL puro) to remove dead cells and bone fragments and to begin selection for engineered cells. RFP fluorescence was confirmed using a Nikon Eclipse Ti fluorescent microscope. After the first passage, selective pressure was maintained with puromycin (RPMI 1640, 10% FBS, 1% pen/strep, and 1 μg/mL puro). Cells were used below passage 6 to prevent phenotypic drift. For more detailed protocol see Appendix 1.

### 2.3 4T1 preparation for caudal artery injection

*In vivo* selected, luciferase-engineered 4T1 cells (4T1-CLL1) were grown to 70% confluence. Cells were trypsinized, washed, and spun down at 300 rcf for 5 min. Cells were then washed in 10 mL of ice-cold PBS, spun down at 300 rcf again, and resuspended in another 10 mL of ice-cold PBS before being gently passed through a 70 μm cell strainer. Cells were counted and then diluted to 5,000 to 50,000 cells/100 μL (depending on number needed to be injected per mouse). Cells were aliquoted into 1.5 mL microcentrifuge tubes and placed on ice before being injected to mice. For more detailed protocol see Appendix 2.

### 2.4 Caudal artery injection

Six-week-old female BALB/cJ mice were fully sedated with a ketamine/xylazine solution (100 mg/Kg and 10 mg/Kg respectively). Mice were placed ventral side up in a cylindrical mouse restrainer and their tails were warmed with a heating lamp. An aliquot of 4T1-CLL1 cells was warmed up in hands and pipetted to mix before being loaded into a 29G½ 300 μL (3/10 cc) diabetes syringe, with a 30 μL air pocket at the base of the plunger. The mouse tail was wiped with ethanol and the tip of the tail pulled straight before inserting the needle (bevel up) 2 cm to 3 cm from tail-tip to enter the caudal artery at a 0° to 10° angle, until a pulse of blood indicated correct position within artery (Supplemental Figure 3, Supplemental Video 1). The plunger was pressed carefully, making sure to feel for any resistance, until 100 μL of cell solution was injected. After injection the needle was held in place for 5 s then rotated 90⁰ before being slowly withdrawn from the artery. A sterile gauze pad was placed with pressure for 60 s to the needle insertion site, to stop bleeding. Mice were placed in a warmed cage and monitored closely prior to their awakening. For detailed protocol, see Appendix 3.

### 2.5 Hematoxylin and eosin staining

Bones were fixed in 10% formalin for 48 h at 4℃ before being decalcified in 14% EDTA, 0.4% PFA pH 7.4 in PBS while shaking at 4℃ for 14 days. Decalcification solution was changed every two days. After decalcification, bones were paraffin embedded and sectioned to 7 μm slices before being mounted on Superfrost slides. Hematoxylin and eosin staining was performed using the standard procedure and slides were mounted in Permount. Slides were imaged using a Nikon Eclipse Ti microscope using a 10x objective.

## 3 Results

**Fig. 1:**
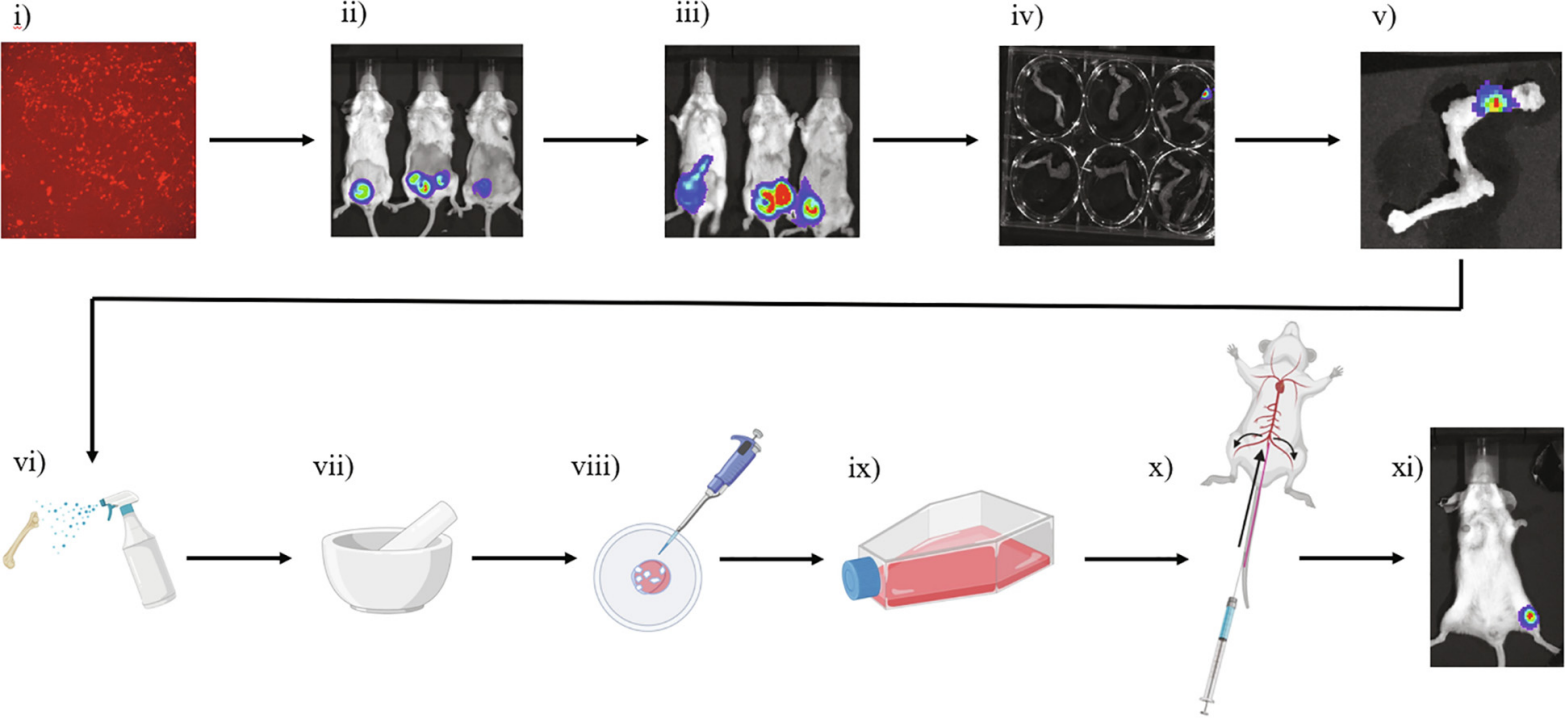
Schematic of method to induce consistent syngeneic bone metastasis. i) Engineer cancer cells to express RFP and luciferase. ii) Inject engineered cells to lower inguinal fat pad of 5-week-old female BALB/c mice. iii) Monitor mice with *in vivo* bioluminescent imaging until fat pad tumor appears to show bone metastasis. iv) Harvest legs of mice and place in a well plate to confirm bone metastasis via *ex vivo* bioluminescent imaging. v) Select bones showing positive *ex vivo* bioluminescent signal. iv) Wash bones with ethanol. vii) Grind up bones with mortar and pestle. viii) Wash ground bones with growth media, pass through a cell strainer to remove bone fragments, then transfer to flask for culture. ix) Culture cells to expand *in vitro*. x) Inject 5 to 8-week-old female BALB/c mice with cultured cells via caudal artery route. xi) Monitor mice with *in vivo* bioluminescent imaging and identify bone metastases. See “Methods” for detailed protocols.

A bone localizing murine breast cancer cell line (4T1-CLL1) was derived from 4T1 cells engineered to express luciferase (Luc) and red fluorescent protein (RFP) by several rounds of *in vivo* selection and used to generate a consistent and robust bone metastasis rate in BALBc mice (Fig. 1). We then compared standard cancer cell injection routes to the recently described caudal artery injection route. The caudal artery injection method combined with 4T1-CLL1 bone localizing cells produced higher rates of bone metastasis and generally lower rates of vital organ metastasis than other standard methods (Table 2).

**Table 2:** Percentage of mice showing 4T1 breast cancer metastases to significant locations for different routes of cancer cell injection.

Metastasis Model	Fat pad	IV	IC	CA 1wk	CA 2wk	CA 1wk (cancer only)	CA 2wk (cancer only)	CA 1wk (PI only)	CA 2 wk (PI only)	CA 2 wk (PI + cancer only)
Number of mice	12	3	3	93	92	68	82	32	31	30
Any cancer development (%)	100	100	100	73.12	89.13	100	100	31.18	96.77	100
Any bone (%)	16.67	66.67	66.67	67.74	85.87	92.65	96.34	29.03	96.77	100
Any leg (%)	16.67	0	33.33	49.46	83.7	67.65	93.9	20.43	93.55	96.67
Spine (%)	0	33.33	66.67	44.09	77.17	60.29	86.59	20.43	93.55	96.67
Pelvis (%)	0	0	66.67	21.51	43.48	29.41	48.78	7.527	0	0
Any fat pad (%)	100	33.33	100	46.24	75	63.24	84.15	18.28	9.68	10
Any vital organ (%)	8.333	100	100	4.301	21.74	5.882	24.39	0	12.9	13.33
Lung (%)	0	100	100	4.301	14.13	5.882	15.85	0	9.68	10
Brain (%)	0	33.33	66.67	0	3.261	0	3.659	0	3.226	3.333
Liver (%)	8.333	33.33	33.33	0	7.609	0	8.537	0	0	0
Kidney (%)	0	33.33	66.67	0	5.435	0	6.098	0	12.9	13.33
2 wk mortality (%)	0	100	100	1.075	1.09	1.471	1.22	1.075	3.226	3.333

Abbreviations: IV: intravenous injection, IC: Intracardiac injection, CA: Caudal artery injection, 1wk: After one week of growth. The “cancer only” group is a subset of the CA group and includes only CA animals that showed any detectable tumor growth up to that point. The “PI” group is a subset of the CA group and includes only CA mice that received perfect injections (100% of cells were delivered into the caudal artery). Any cancer development refers to the tumor take rate for the specified group. The Intravenous model established with a single injection to the tail vein with 500,000 Luc-RFP cells and bioluminescent imaging performed 11 days post cell injection, the Intracardiac model established by injection to left ventricle of 200,000 Luc-RFP cells and bioluminescent imaging performed 11 days post-injection, the Fat pad model established by subcutaneous injection on top of one or both of the lower inguinal mammary fat pads (Supplemental Fig. 1a “#5”) of 100,000 Luc-RFP cells mixed with 50% Matrigel™ and bioluminescent imaging performed 14 days post cell injection, the Caudal artery (1 wk) model established by intra-arterial injections of 10,000 to 20,000 Luc-RFP cells imaged after 7 days post cell injection, Caudal artery (2 wk) model contains the same animals as “Caudal artery (1 wk)” and bioluminescent imaging was performed 14 days post cell injection. Bioluminescent images (front and back) were exposed for 1 s, 60 s, and auto exposed to get high sensitivity for weak signals and minimize saturation caused by strong signals.

### 3.1 *In vivo* selection for bone localizing 4T1 cells

**Fig. 2:**
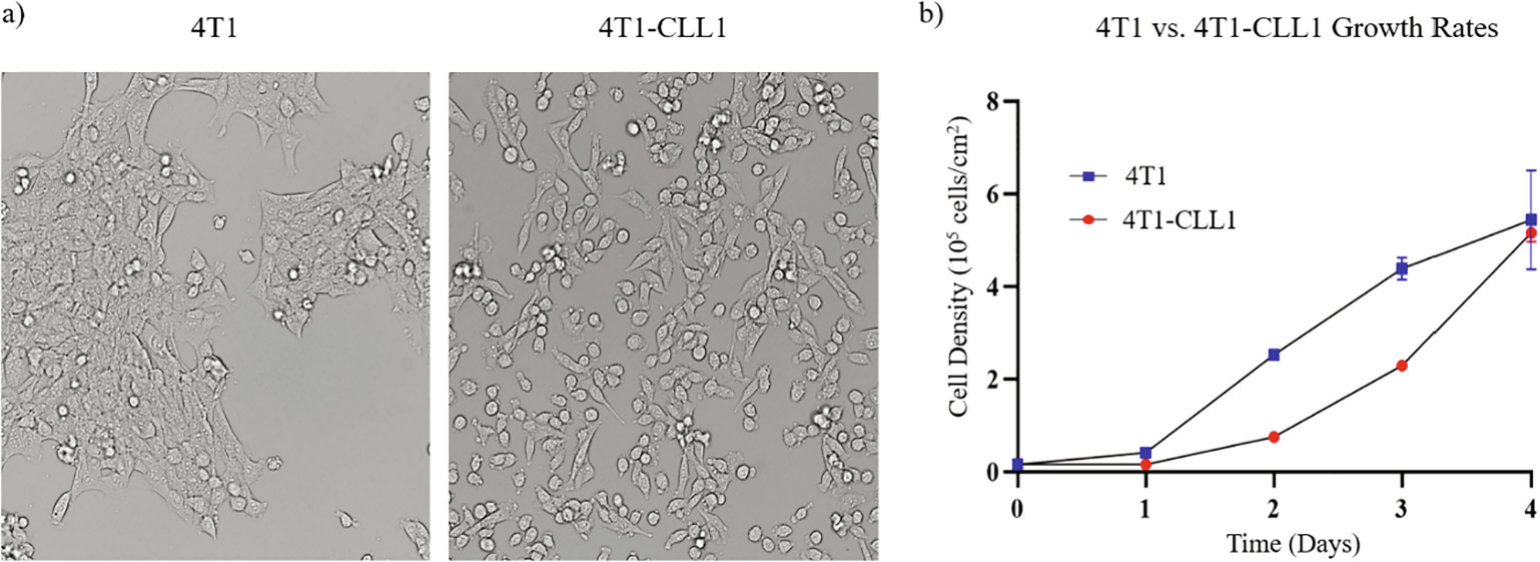
The 4T1-CLL1 line shows some phenotypic differences to 4T1 cells. a) Morphological comparison between the two cell lines at equivalent densities shows the 4T1-CLL1 cells appeared to transition from a clumped colony forming behavior to a migratory phenotype. b) To investigate possible changes in growth rate, 4T1 (P15) and 4T1-CLL1 cells were plated in triplicates, staring at 5,000 cells per well (96 well plate) and grown using Roswell Park Memorial Institute (RPMI) 1640 Medium (supplemented with 10% FBS). Cell density was recorded by hemocytometer and the mean of triplicates (±SEM) for each cell line was calculated every 24 h.

One important aspect of many murine cancer studies is the ability to track cancer growth *in vivo*. Using lentiviral particles (GenTarget Inc. LVP324), 4T1 cells were engineered to express RFP and luciferase, which enables tracking of cancer cells both *in vivo* and within post-mortem tissues (Fig. 1i). While 4T1 cells can produce bone metastases, they also frequently create primary tumors in non-bone organs, particularly the mammary fat pads. To increase the rate of bone specific metastases, we used *in vivo* selection. Two rounds of *in vivo* selection were used to produce a 4T1 derivative cell line “4T1-CLL1” (Fig. 1ii-1ix), which we have shown can generate bone metastasis at high rates when used in combination with a caudal artery injection, in BALB/cJ mice (Table 2). The 4T1-CLL1 cell line exhibited similar growth rates when compared to 4T1 cells (Fig. 2b). However, we did note an interesting shift in morphology within the 4T1-CLL1 cells, characterized by a tendency to spread into the empty spaces of the dish, rather than grow in compact colonies (Fig. 2a).

**Fig. 3:**
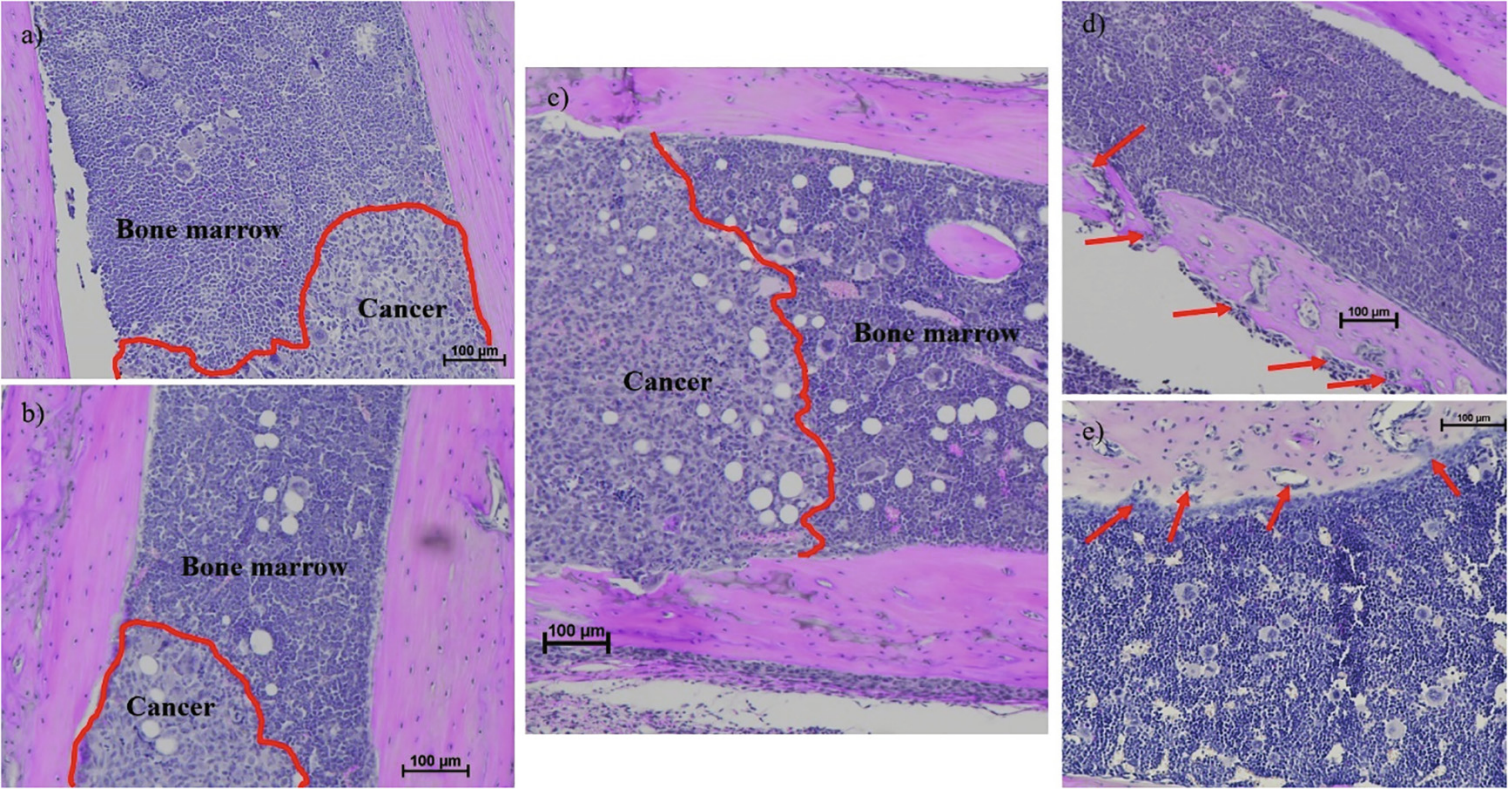
4T1-CLL1 delivered via caudal artery produce invasive and destructive bone metastases. a), b), and c) show the invasion of cancer cells into the bone marrow of mouse femurs. The red line indicates the front of the tumor invasion. d) and e) show degradation of bone and simultaneous invasion of tumors cells (red arrows). Tumors developed after 10,000 to 20,000 4T1-CLL1 were delivered via caudal artery to BALB/cJ mice and allowed to grow for two weeks before being sacrificed for histological analysis. (For interpretation of the references to colour in this figure legend, the reader is referred to the web version of this article.)

The 4T1-CLL1 cell line was found to be extremely aggressive when delivered via the caudal artery to BALB/cJ mice (Fig. 3). After only two weeks, leg bones received significant damage due to the osteolytic ability of the cells. We have previously shown the severe bone erosion this model produces via microCT scans [12]. 4T1-CLL1 cells (1 × 104) delivered by caudal artery produced extensive shaft and epiphysis damage. Bone metastases significantly reduced overall femur bone volumes and reduced trabecular bone in the epiphysis. While invasion into the bone marrow was frequent (Fig. 3a,b,c), the majority of the tumor mass was concentrated in the epiphysis [12]. Bones were frequently so damaged and broken by tumor invasions that neither histology nor microCT were possible.

### 3.2 Comparison of caudal artery injection route to other standard bone metastasis inducing cell delivery methods

**Fig. 4:**
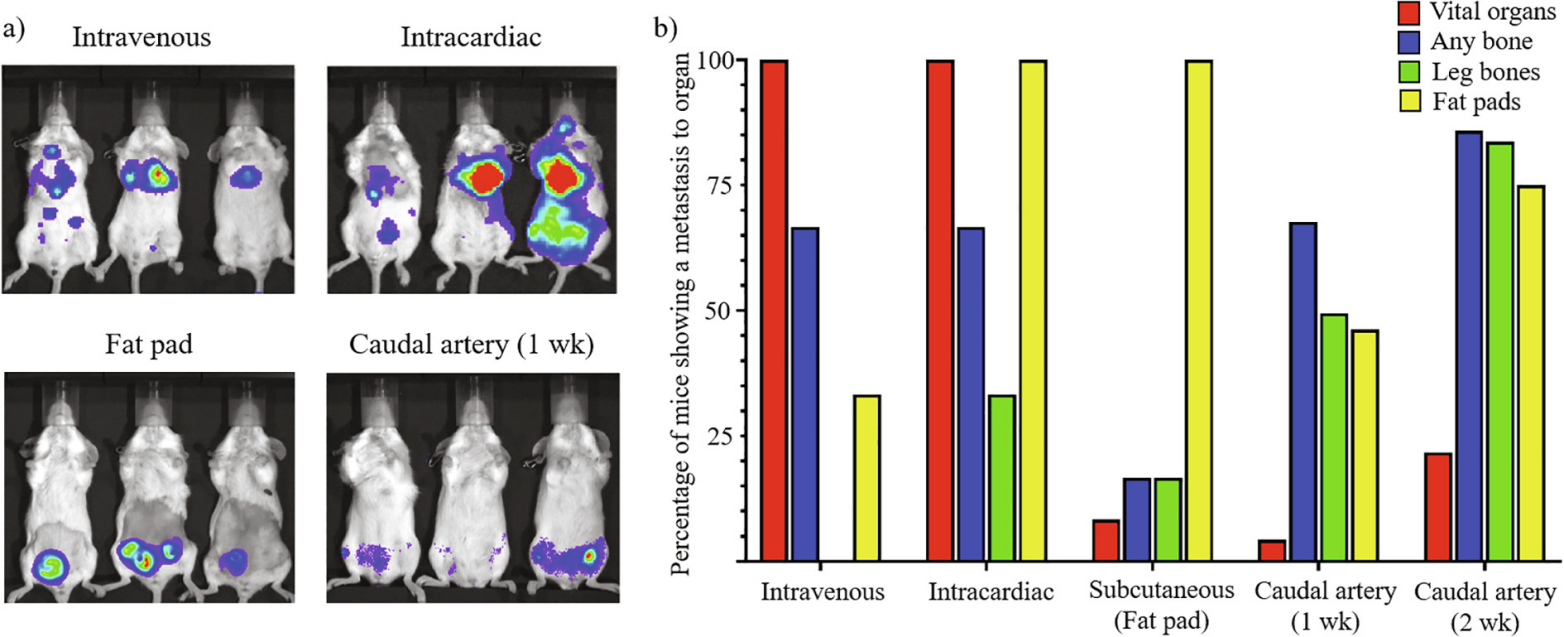
Graphical comparison of 4T1 breast cancer metastasis locations for different routes of cancer cell injection. a) Representative images of tumor growth for each route after one to two weeks post cell transplantation. b) Comparison of metastasis location for each Luc-RFP-4T1-CLL1 cell implantation route. The “Vital organs” were counted if a mouse had at least one clear tumor in the brain, lungs, heart, liver, and/or kidneys. The “Any bones” were counted as any signals that were determined to be in bones, including the femur and tibia/fibula leg bones, lower spine, pelvis, and ribs. The “Fat pads” included any tumors at the region of the abdominal mammary fat pads. The percentage of mice showing metastasis to organs was calculated by counting the number of animals showing bioluminescent signal in at least one of the organs in each group, divided by the total number of animals in the experiment and multiplying by 100. Cell quantities injected for each route, and the methods are described in more detail in Table 2.

The standard routes of injecting cancer cells to produce bone metastasis (intravenous, intracardiac, subcutaneous mammary fat pad) were insufficient for our previous bone metastasis mouse studies, because they lacked speed, consistency, low mouse mortality (vital organ metastases), and high rates of bone metastasis. Mice injected with 4T1 by intravenous (IV) or intracardiac routes (IC), rapidly became unhealthy and died (Table 2). Bioluminescent imaging revealed large tumors in the lungs, intrapleural cavity, and/or heart for both IV and IC injection routes (Fig. 4a). A high rate of mortality creates significant problems with consistency in a bone metastasis study, because animals die before bone metastasis or before bone metastasis growth can be sufficiently evaluated. Fat pad injections are used frequently for triple negative breast cancer and patient derived xenograft models and used to study cancer cell intravasation and dissemination. Subcutaneous mammary fat pad injections produced low rates of mortality, but had low rates of bone metastasis, took a long time to metastasize to bones, and/or were difficult to evaluate on a bioluminescent basis, due to the saturating signal produced by the large fat pad tumors (Fig. 4a). The saturation of the primary tumors can be alleviated by surgical resection of the primary tumor, which can also result in even fewer vital organ metastases. However, tumor resections are invasive and can result in a significant increase in animal pain, which is avoided by use of the caudal artery route. The caudal artery injection route produced low rates of vital organ metastasis, high rates and of leg bone metastasis in only one to two weeks, and fat pad signal saturation was far less an issue (allowed identification of small bone metastases close to the fat pads) than was the case for a direct fat pad injection (Fig. 4). Additionally, the ability to see the injection fluid traveling through the vessel after caudal artery injections, allows stringent judgement of precision compared to intracardiac injections, in which the actual injection is not seen. A comparison of the four injection routes, shows similar rates of bone metastasis between intravenous, intracardiac, and caudal artery mice, but vital organ metastasis rates closer to those of the fat pad mice (Fig. 4b). Significantly, for our study, leg bone metastasis rates were higher for caudal artery injection mice than any other route (Fig. 4b). There were significant numbers of mice with tumors clearly localized in the lower inguinal fat pad regions for all models tested with 4T1 cell lines (Fig. 4).

### 3.3 Optimizing 4T1-CLL1 caudal artery injection for desired growth rates and consistency

**Fig. 5:**
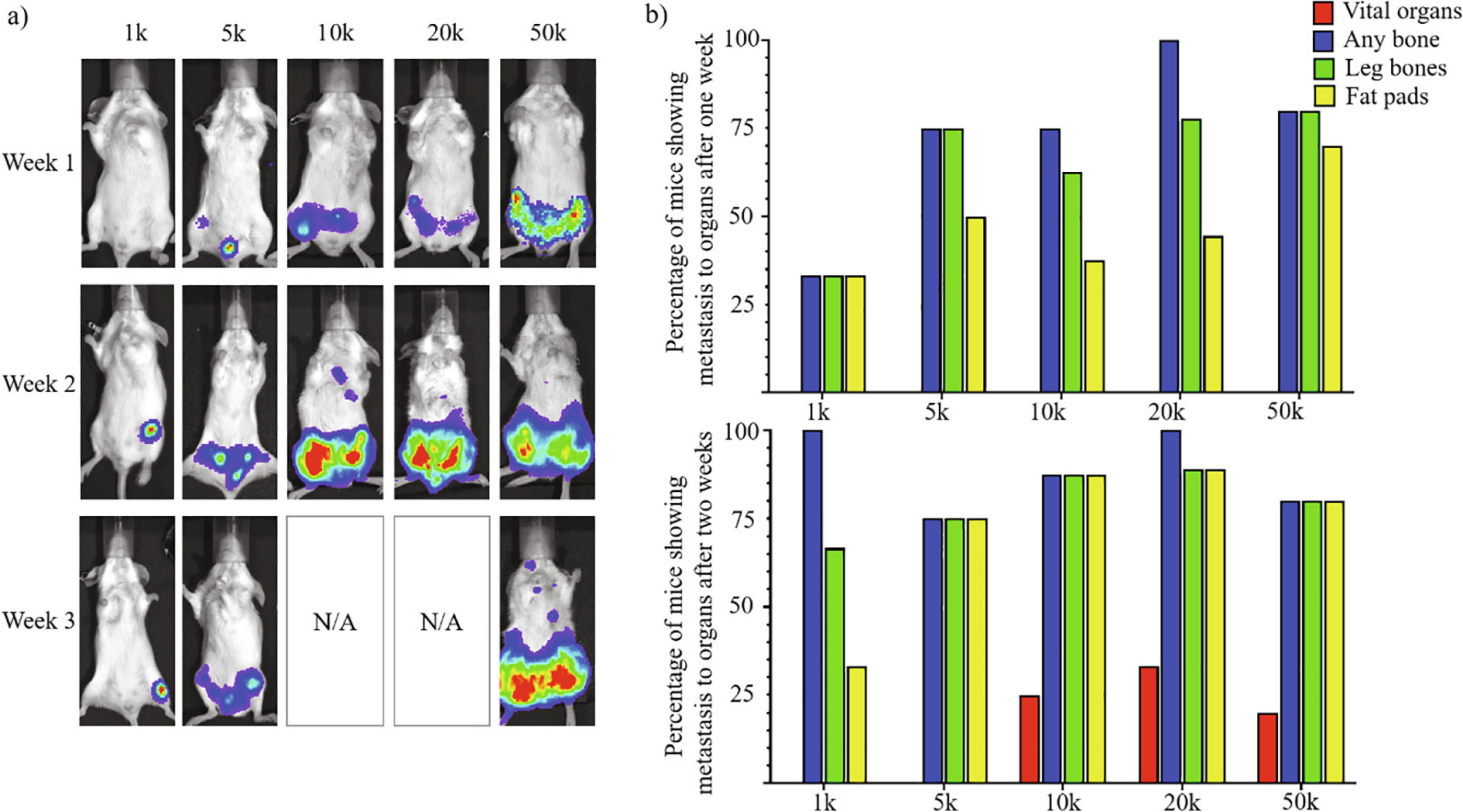
Quantity of cells injected alters pattern of 4T1-CLL1 metastasis. a) 4T1-CLL1 cells were injected via caudal artery and allowed to grow for three weeks (the 10 k and 20 k mouse groups were sacrificed, as part of an experiment, before a week three imaging could be performed). Bioluminescent imaging was performed biweekly to monitor tumor growth over time using automatic exposure and have automatic colorimetric scaling (non-quantitative). Images for each cell quantity group are representative of the overall progression of each group of mice over time. b) Comparison of metastasis locations for various cell quantities injected via caudal artery after one week (top) and two weeks (bottom) of growth. The “Vital organs” were counted if a mouse had at least one clear tumor in the brain, lungs, heart, liver, and/or kidneys. The “Any bone” were counted as any signals that were determined to be in bones, including the femur and tibia/fibula leg bones, lower spine, pelvis, and ribs. The “Fat pads” included any tumors at the region of the abdominal mammary fat pads. The percentage of mice showing metastasis to organs was calculated by counting the number of animals showing bioluminescent signal in at least one of the organs in each group, divided by the total number of animals in the experiment and multiplying by 100. Number of animals included: 1 k n = 3, 5 k n = 4, 10 k n = 8, 20 k n = 9, and 50 k n = 10.

Determining the appropriate cell number to inject is important, as it has a major impact on tumor growth rates [13], [22], [23]. Due to the high bone delivery rate of the caudal artery injection, and the aggression of the 4T1 cell line, few cells are needed to generate detectable tumors, when compared to xenogeneic cell lines. The 4T1 cell line is so aggressive, that it causes mouse morbidity with off-target metastases (such as to the vital organs) in other injection routes. However, most of the growth from caudal artery delivered 4T1-CLL1 cells is localized in the bones (spine, leg bones, and pelvis) and/or the inguinal mammary fat pads (Table 2, Fig. 4a, Fig. 5). Injection of a lower number of cells (e.g., 1,000) can result in very specific bone metastasis, but the time it takes for the tumors to become detectable/trackable can vary (1 to 6 weeks). Since a few of the mice did not develop tumors within 6 weeks, the number of mice with bone metastases available for the experiment can be unclear. While the mice injected with 1,000 cells developed some of the cleanest bone metastases (minimal metastases outside leg bones), these metastases also appeared to be inconsistent in their growth rates, since some mice developed detectable tumors in much less time than others. This large baseline variation in growth can make comparison between treatment groups far more difficult. Depending on what the metastasis model is meant to evaluate, the growth rate can be controlled by injecting more cells (Fig. 5). If many more cells are injected (e.g., 50,000), the rate of detectable bone tumor development becomes fast and consistent (5 to 6 days). However, the rapid growth of resulting tumors may require sacrificing the mice after only 2 weeks [24]. The ability to evaluate therapeutics may also be hindered by such a high rate of tumor growth, since even a high dose of standard chemotherapeutic drug (5-fluorouracil) was unable to sufficiently inhibit tumor growth (Supplemental Fig. 4). Therefore, a reasonable balance between tumor growth rate and consistency seems to be 5,000 cells injected, but this will depend on the desired metastasis characteristics (ex. Consistency between animals, rate of tumor growth, and whether a leg bone specific tumor is required). A 5,000-cell caudal artery injection will produce a detectable tumor consistently within 2 weeks, if the injection itself is done correctly and the cells are healthy (Fig. 5).

Interestingly, in an experiment where 32 mice received perfect injections (all cells delivered to caudal artery in first attempt) of 10,000 to 20,000 4T1-CLL1 cells, excluding one mouse which did not develop any detectable cancer, we achieved a 100% leg bone metastasis rate within 2 weeks (Table 2). Of these mice, only 13.33% developed metastases to vital organs within two weeks, most of which appeared to be in the lungs, liver, and kidneys (Table 2). The relatively low rate of vital organ metastasis limits morbidity in mice and allows a focus on bone tumors and their treatment. If the cells are not delivered perfectly, for example if some cells are delivered subcutaneously while attempting to insert the needle to the caudal artery, the rates of vital organ metastasis increase with each failed injection, most likely caused by the proximity of tail veins to the caudal artery (Supplemental Fig. 1b) and the high motility of the 4T1-CLL1 cells (Fig. 2).

## 4 Discussion

Several factors significantly affect the success rate of injections. It is important to create detailed standard operating procedures for cell preparations, to minimize technique variations between different personnel, because these variations can cause different tumor growth rates. Mice should be sedated with a ketamine/xylazine solution, which has a greater effect on hemodynamics (including arterial pressure). Appropriate sedation is required so that injected cells can flow against arterial pressure until they reach the branching point of the iliac arteries and can flow toward the leg bones [25]. Adequate heating of the tail prior to injection (dilates caudal artery), and sufficient injection training of personnel is essential to getting perfect injections, thus minimizing vital organ metastases, while maximizing leg bone metastases.

One characteristic of the 4T1 cell line noticed in all experiments, was a tendency to form tumors at the mammary fat pad tissue beneath nipples #4 and #5 (Supplemental Fig. 1a). While there were a high percentage of tumors in bones, many were also observed at these fat pads (Fig. 4b). This is likely because the 4T1 cell line originates from mammary gland tissue, and that the caudal artery injection route delivers cells to vessels which feed the abdominal mammary glands [26]. Further rounds of *in vivo* selection might increase the propensity of the cells for bones over fat pads. Additionally, further studies should be done to characterize the 4T1-CLL1 cell line to determine what molecular alterations result in better osteotropism and to confirm maintenance of osteomimicry and osteotropism after *in vitro* passaging. It is important to minimize the number of *in vitro* passages for cell lines which have undergone *in vivo* selection, because they may lose some of their selected phenotype.

From the primary sites of tumor engraftment (usually bones and fat pads) the cells can further metastasize to any other location including vital organs but appeared to spread to neighboring organs more frequently than distant ones (Supplemental Figure  1c). The longer animals were kept alive, the more vital organ metastases were detected. This is consistent with other routes of injection, except that other routes produced vital organ metastases much sooner after cells were injected. The preference for fat pad tumor growth makes this model similar to direct subcutaneous fat pad injection route, except that the resulting fat pad tumors in this model are relatively small and usually do not mask other sites of metastasis.

There are still several caveats to address with this bone metastasis model. The frequency of spine tumors produced high rates of lower body paralysis in mice with large tumors after two weeks, but this can also happen at high occurrence in other models of bone metastasis [27], [28], [29]. This can be mitigated by using a minimal cell injection numbers, that will slow overall tumor growth. As mentioned above, the precision of the injection and the quality of the cells are important to model consistency. In large experiments, where many mice require caudal artery injections within a short time period, the time constraints will increase the chance for mistakes and lower the ratio of “perfect injections”. Time constraints are an issue particularly because cells being used for animal injections should not be left sitting on ice for long, because it may reduce their health, viability, and cause cell clumping. While the percentage of “perfect injections” above may belie the ease of the technique, we defined a “perfect injection” strictly, such that repositioning of a needle after the first insertion (despite delivering cells directly to the caudal artery), was a disqualifier. In smaller experiments (under 20 mice), “perfect injections” can be completed in almost every mouse.

Another consideration is that while this model allows tracking tumor growth in live animals, determination of precise metastasis locations can be difficult, without sacrificing the animal and imaging the organs *ex vivo*. Unfortunately, in large animal studies, *ex vivo* imaging can be impractical because it requires time which might be used to preserve sensitive tissues for downstream assays (ex. FACS on bone marrow and spleen). The *in vivo* bioluminescent imaging method we mentioned above (Supplemental Figure 5) uses two-dimensional images and requires us to estimate the actual position of a tumor in three-dimensional space and determining if a tumor is inside or outside of a bone can be difficult. To mitigate this issue, we performed *ex vivo* bioluminescence imaging in several studies to confirm our ability to determine if metastases were within the bone (Supplemental Figure 2). Additionally, we made use of multiple exposure times, which minimizes masking by saturating tumors in later stages of experiment, while also allowing detection of small tumors in early stages. Front and back images can be taken to show whether the signal is more ventral or dorsal and thus increase metastasis tracking accuracy. Some tumor positions can be better identified by the signal strength of front (ventral) images, relative to the back (dorsal). For example, if in the spine, the signal will be stronger on the back image than the front, but the position of the signal along the midline of the mouse will be consistent.

## 5 Conclusions

Due to the limitations of other injection routes, the caudal artery delivery method described by Kuchimaru et al. should become the new standard of delivering cells to the bones via the circulatory system. We described, in more detail, a simple and effective way to generate syngeneic breast cancer bone metastasis in a mouse model. For perfectly executed caudal artery model inductions, we were able to get bone metastasis rates over 95%. These metastases were not only consistent in their sizes, but also in their locations. There were few metastases to vital organs, which further improved the experimental consistency, since mice maintained their health longer and did not need sacrificing before the planned experimental endpoint. The consistency of this model allows a reduction in both the total number of animals used, and the costs relating to syngeneic bone metastasis studies. Consistency also increases the robustness of therapeutic studies, because it allows more homogenous grouping of experimental animals.

## Funding

This work was supported by the NIH (R21CA219225 to W.Z.), the 10.13039/100000005DOD (W81XWH-17-1-0522 to W.Z.), and a contract with Baylx Inc. (BI-206512). H.P.F. was supported by the 10.13039/100000065National Institute of Neurological Disorders and Stroke of the NIH (T32NS082174). A.I.S was supported by the 10.13039/501100004097Fondation ARC pour la recherche sur le cancer (SAE20150602901), and a contract with Amberstone Biosciences Inc., and L.L. was supported by Baylx Inc. (BI-206512). The project described was also supported by the 10.13039/100000097National Center for Research Resources and the 10.13039/100006108National Center for Advancing Translational Sciences, 10.13039/100000002National Institutes of Health and 10.13039/100000054National Cancer Institute of the 10.13039/100000002National Institutes of Health under award number P30CA062203. The content of this paper is solely the responsibility of the authors and does not necessarily represent the official views of the 10.13039/100000002National Institutes of Health. The funders above did not have any involvement in the study design, data collection, data analysis, interpretation, or manuscript writing.

## Appendix

Highly detailed protocols for establishing syngeneic bone metastasis.

## Appendix 1 *. In vivo* bone metastatic cell selection

*Note: In vivo selection via a mammary fat pad primary tumor is described here, but caudal artery delivery of the primary tumor might produce osteotropic cell phenotypes with cleaner osteomimicry.*

1) Transduce 4T1 cells to express RFP and luciferase using a multiplicity of infection of 10 (see manufacturer protocol).

*Note: A stable RFP/Luc cell line should be generated by several passages of selective pressure (*1 μ*g/mL puromycin in growth media). FACS for the strongest RFP signal cells, followed by expansion under selective pressure (*1 μ*g/mL puromycin in growth media). Cells should also be validated by a luciferase activity assay using a* 7.5 μ*g/mL solution of D-luciferin in PBS (see manufacturer protocols for D-luciferin stock solution preparation and luciferase activity assay). For murine cell lines, use of an EF1-alpha promoter in place of CMV can reduce methylation and silencing of the RFP/Luc genes during in vitro passages.*

2) Prepare Matrigel™ by thawing frozen aliquots on ice at 4 °C overnight.
3) Trypsinize engineered cells, neutralize trypsin by adding media, spin down at 300 rcf, and resuspend in ice cold RPMI 1640 media (no FBS) at a concentration of 2 million cells/mL.
4) Gently mix cell suspension with ice cold Matrigel™ in a 1:1 ratio (final cell concentration of 1million cells/mL).

*Note: Recommended to prepare 50% more cell/Matrigel*™ *solution than is needed to account for loss in syringe.*

5) Place cell/Matrigel™ solution on ice and inject as soon as possible.
6) Fully sedate 6-week-old female BALB/cJ mouse (e.g., ketamine injection or isoflurane nose cone).
7) Shave mouse abdomen so that the lower inguinal mammary fat pads are clearly visible (see Supplemental Figure  1a).
8) Wipe the lower abdomen with 70% ethanol.
9) Place the mouse on a clean flat surface with arms taped down outstretched.
10) Using tweezers, lift the nipple covering one of the lower inguinal mammary fat pads and perform subcutaneous injection at 100,000 cells per mouse (100 μL of cell/Matrigel™ solution).
11) Monitor mice for tumor growth on a biweekly basis. This can be done by IP injection of 150 mg D-luciferin/Kg of mouse body weight, and imaging isoflurane nose cone sedated mice 10 min post-injection using an IVIS Lumina Animal Imager. Taking a front and back image of mice enables a more accurate identification of tumor locations and increasing exposure times can help to detect weak signals in the early stages of tumor growth.

*Note: The fat pad tumors need to be covered with light absorbing black paper and/or tape to minimize saturation of the detector*.

12) When a mouse with a robust detectable leg bone metastasis is identified, immediately sacrifice it in a biosafety cabinet and use sterile surgical tools to remove the leg bones showing signal. This step must be performed quickly, as the bioluminescence of the engineered tumor cells will decrease significantly over time after 20 min post D-luciferin injection.

*Note: Be careful not to break the bones, because it will expose cells to contamination and ethanol washes, which may kill cells*.

13) Place the leg bone in a well plate submerged in sterile PBS and close the lid.
14) Use IVIS Lumina to image leg bones *ex vivo*. It is important to confirm that the *in vivo* bioluminescent signal is coming from the bone (Supplemental Figure 2).
15) After *ex vivo* confirmation that the tumor is inside bone, transfer well plate back to biosafety cabinet.
16) Carefully remove all muscle tissue without damaging the bones.
17) Place the intact leg bones in a well plate filled with 70% ethanol for about 10 s.
18) Rinse off the excess ethanol by submerging the intact leg bones in a well plate filled with sterile PBS.
19) Using a sterile mortar and pestle, grind the leg bones into fine pieces around 1 mm in diameter.

*Note: Excessive grinding of bones can harm cell viability*.

20) Use 3 mL of growth media (RPMI 1640 containing 10% FBS, and 1% pen/strep) to rinse both the pestle and the sides of the mortar.

*Note: Optional collagenase digestion can be performed on bone fragments but is not necessary*.

21) Aspirate the growth media and filter it through a 70 μm cell strainer into a clean 50 mL tube.
22) Repeat steps 19–20.
23) Pipette the filtered media into a T25 flask (or equivalent petri dish). Do not transfer any remaining bone fragments.
24) Change media the following day to remove dead cells and bone fragments (use RPMI 1640 containing 10% FBS, 1% pen/strep, and 3 μg/mL puro).

*Note: The construct containing the RFP/Luciferase gene specified also contained puro resistance, but if another construct is used, confirm it has the puro resistance gene.*

25) Over the following days monitor cell growth and check RFP fluorescence to confirm cells present are indeed the engineered tumor cells.
26) After the first passage, maintain selective pressure using RPMI 1640 containing 10% FBS, 1% pen/strep, and 1 μg/mL puro. Cell lines should be used at low passage numbers to prevent phenotypic drifting (ex. Below passage 6).

## Appendix 2 . 4T1 Preparation for caudal artery injection

*Note: Unless otherwise specified, any liquids added to (or removed from) cells should be done at the side of the flask/dish, to prevent disrupting the cell layer. All pipetting should be done at low velocity to prevent harm to cells. Volumes specified are intended for T175 flasks but may need adjusting if alternate culture containers are used.*

1) Visually inspect the cells to check their confluence (should be at 70–80% confluence).
2) Aspirate the cell growth media.
3) Wash the cells with 15 mL PBS by gently tilting flask/plate back and forth briefly after addition.
4) Aspirate the PBS.
5) Add 1 mL trypsin and place in 37 °C incubator for 3 min.
6) Check cells under a bright field microscope to make sure they are detaching.

*Note: Leaving cells in trypsin for too long will lead to cell death and the DNA released from dead cells will stick cells together to form clumps*.

7) Neutralize the trypsin with 9 mL cell growth media and wash the surface of flask gently, by pipetting the full volume of media over one third of the flask at a time (total of three washes), to detach cells from flask/plate.
8) Transfer the cell suspension to a 50 mL conical tube.
9) Centrifuge the cells at 300 rcf for 10 min at 4 °C.
10) Aspirate the media, making sure not to disrupt cell pellet.
11) Gently resuspend the cells in 1 mL ice-cold PBS (using a P1000 pipette) to break up the pellet, then add an additional 9 mL of ice-cold PBS to wash the cells of remaining FBS proteins.
12) Take a small aliquot of the cell suspension for counting.
13) Centrifuge the cells at 300 rcf for 10 min at 4 °C.
14) Count the cells while waiting for centrifugation.
15) Resuspend the cells with an adequate amount of ice cold PBS (10 mL).

*Note: Carefully break the pellet first by mild bumping against a grate, gently pipette up and down a few times with a 10 mL serological pipette and then draw up entire solution to be strained.*

16) Filter the cells through a 70 μm cell strainer.
17) Count filtered cells using a hemocytometer to confirm exact cell concentration.
18) Dilute the cell suspension to 50,000 cells/mL.

*Note: If more or less than 5,000 cells per mouse is desired, then adjust the concentration so that 100 μL will contain that number of cells.*

19) Recount to confirm exact concentration and volume needed to inject mice with 5,000 cells per injection (∼100 μL).

Note: Check for a single cell suspension using hemocytometer and microscope.

20) Aliquot cells at 150 to 500 μL per 1.7 mL tube (minimize the number of mice that will receive cells from a single aliquot), then place cells on ice.

*Note: Since the cells will be mixed by pipette prior to each injection, separation into aliquots minimizes exposure to shear forces of pipetting.*

21) Inject cells within 1 h of preparation.

*Note: Cell viability drops over time, but if prepared properly cells can last at least 3 h on ice at > 85% viability.*

## Appendix 3 . Caudal artery injection

*Note: Prior to set up clean all surfaces with 70% ethanol and make sure all materials are ready to use.*

1) Label each mouse with ear tags for accurate monitoring of individual mice.
2) Take and record the body weight of each mouse and calculate the amount of ketamine/xylazine solution needed to sedate each mouse (*e.g*., 100 mg/Kg and 10 mg/Kg of mouse weight).

*Note: The potency of the ketamine/xylazine solution is important, because it affects the blood flow rate, and thus the ability of cells to arrive at the leg bones.*

3) Sedate mice with an IP injection of ketamine and xylazine solution using a 27G½ needle.

*Note: It is best to sedate only a few mice at a time, so that they are not sedated long while waiting for their caudal artery injection. Mice lose body heat rapidly and sedation without heating can induce hypothermia and an exaggeration of sedative effects (may result in mouse death).*

4) Slide the mouse ventral side up into a cylindrical mouse restrainer to hold the mouse in a convenient position for injection.
5) Warm each mouse tail with either a heating lamp or warm sterile water. This dilates the blood vessel and makes the injection easier.

*Note: Lamp should be at least 30 cm from the mouse to prevent overheating. Overheating can result in the development of necrosis in the tail in the following days.*

6) Warm the cell aliquot up by holding in hand for 2 min.
7) Clean the mouse tail by wiping it with a 70% ethanol wetted cotton gauze pad.
8) Gently pipette the cell aliquot up and down to make into a single cell suspension distributed homogenously in solution.

*Note: It is important to complete the injection within a few minutes of loading the syringe to prevent cell sedimentation. If cells were not prepared carefully enough, they may form visible clumps which should not be injected to the mouse.*

9) Load a 29G½ 300 μL (3/10 cc) diabetes syringe with the cells, making sure to leave a 30 μL air pocket at the base of the plunger. The air pocket enables you to see a pulse of blood into the syringe, which indicates when you have entered the blood vessel.

*Note: Do not invert the syringe roughly or the air pocket will be dislodged from the base of the syringe.*

10) Eject any bubbles near the tip of the needle until liquid is visible.
11) If necessary, prepare the next group of mice for sedation so that they are ready to inject after completing the first round of injections.
12) Gently pull the tip of the tail straight with one hand and hold it extended while injecting (position syringe so that bevel is facing up during injection) with the other hand.

*Note: It is important to keep your hand steady after the needle is in the vessel to prevent the needle from moving and causing a subcutaneous injection.*

13) Slide the needle into the bottom of the mouse tail slowly, 2 cm to 3 cm from tip to enter the caudal artery at a 0° to 10° angle, until a pulse of blood indicates correct positioning within the artery (Supplemental Figure 3, Supplemental Video 1).

*Note: As with a tail vein injection, resistance to the injection indicates the needle is not positioned within the vessel and will be subcutaneous if injected.*

14) Move the needle forward 3–5 mm to make sure it is well within the artery before depressing the plunger.
15) Inject 100 μL of cell solution by pressing the plunger smoothly at a rate of 30 μL per second.

*Note: Since a partial subcutaneous injection will interfere with imaging and create inconsistent tumor sizes, avoid injecting subcutaneously and move proximally 1 cm down the tail to a new injection site if not in the vessel on first attempt.*

*Note: Do not attempt further injections beyond 3 failed attempts, as this will damage the tail and prevent future injection attempts. Mice can be reinjected after a two-day healing period.*

16) After injection, do not withdraw immediately and instead pause for 5 s and rotate the needle gently 90⁰ before withdrawing needle. This helps to diminish the backward pull on injected cells that may interfere with their flow to the target organs.
17) Withdraw the needle and dispose in sharp’s container.

*Note: Do not use the same needle/syringe for multiple mouse injections, because the tip will be slightly blunted and cells from the solution/blood may clog the needle.*

18) Place a sterile gauze pad over the injection site and apply pressure for 60 s to stop bleeding.
19) Place mice in a clean cage on a heating mat (prevents hypothermia) and monitor until they wake up. Mice should be checked for abnormal behavior that might indicate a pulmonary embolism.

*Note: At the recommended concentration of ketamine + xylazine, the mice will normally wake up 30–60 min, post sedation.*

20) Check on the injected mice the following day to make sure they are healthy and that there is no injury to the tail.
