# Supplementary material for: Optimization of a syngeneic murine model of bone metastasis
Source: J Bone Oncol. 2020 May 31;23:100298. doi: 10.1016/j.jbo.2020.100298 (PMC7334391; doi:10.1016/j.jbo.2020.100298)
Supplement: Supplementary data 1 [file mmc1.docx]

**Supplementary Materials**


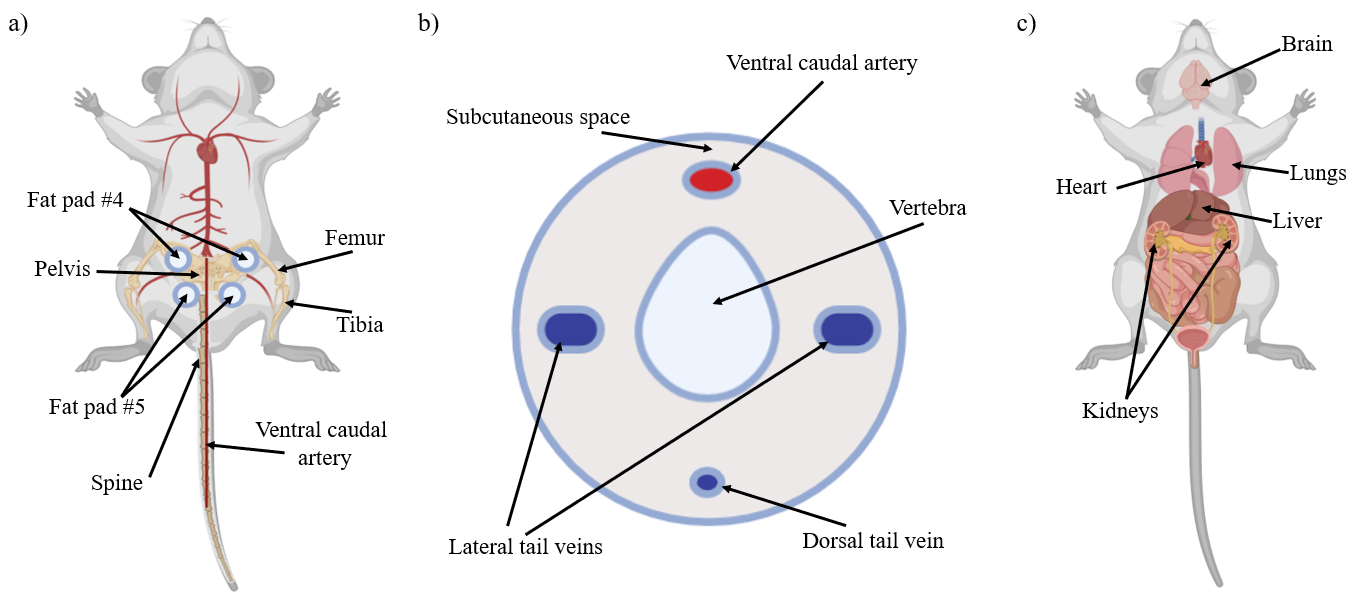


**Supplemental Figure 1.** Important landmarks in mice for a 4T1 caudal artery bone metastasis model. a) Important landmarks for primary metastases. b) Cross section view of mouse tail and blood vessels. c) Vital organ landmarks relating to 4T1 metastasis.


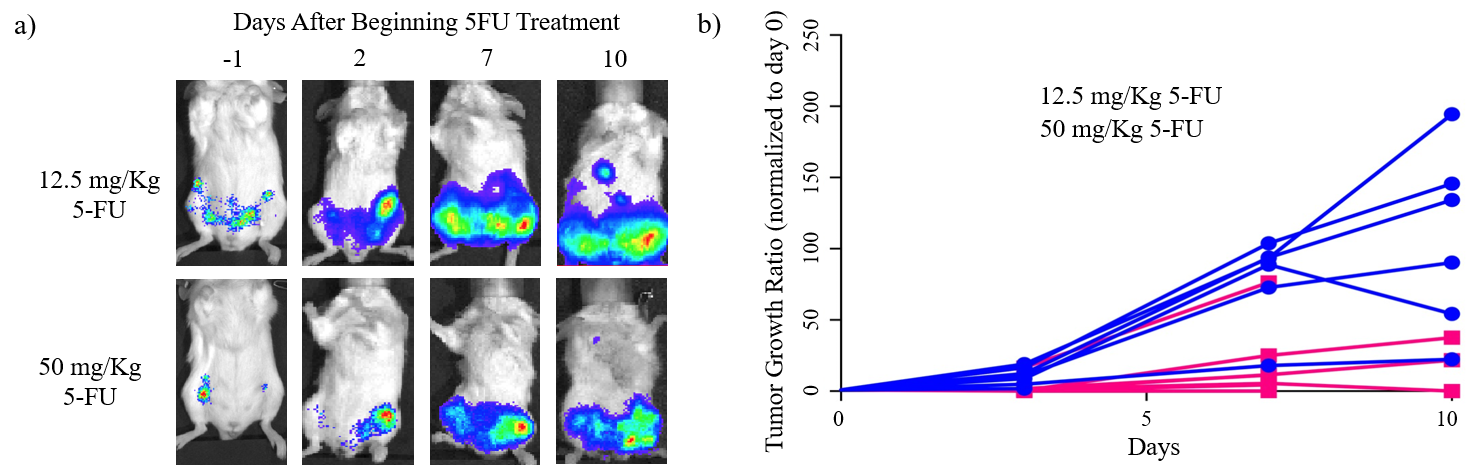


**Supplemental Figure 2.** 4T1-CLL1 tumor growth over time is not sufficiently inhibited by 5-fluorouracil. a) Representative images showing the progression of tumors for two groups of mice receiving separate doses of 5-fluorouracil (5-FU) from one day prior to treatment, to 10 days after the first treatment injection. The 4T1-CLL1 cells were delivered via the caudal artery (5 x 10^4^) 5 days prior to beginning of 5-FU treatment. The 5-FU Treatments were suspended in PBS and given via IP injection daily from day 0 until day 5. b) Tumor growth of each mouse treated by either 12.5 mg/Kg or 50 mg/Kg quantified by bioluminescent signal using the entire mouse as a ROI.


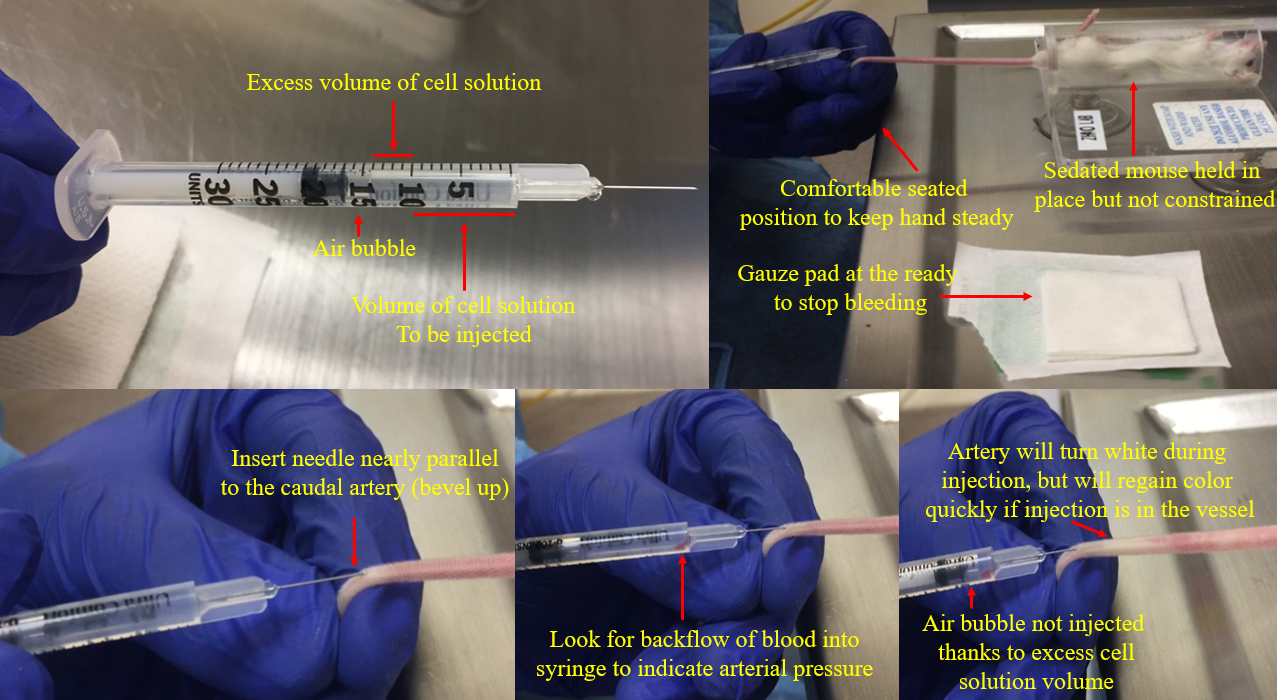


**Supplemental Figure 3.** Demonstration of caudal artery injection with comments.


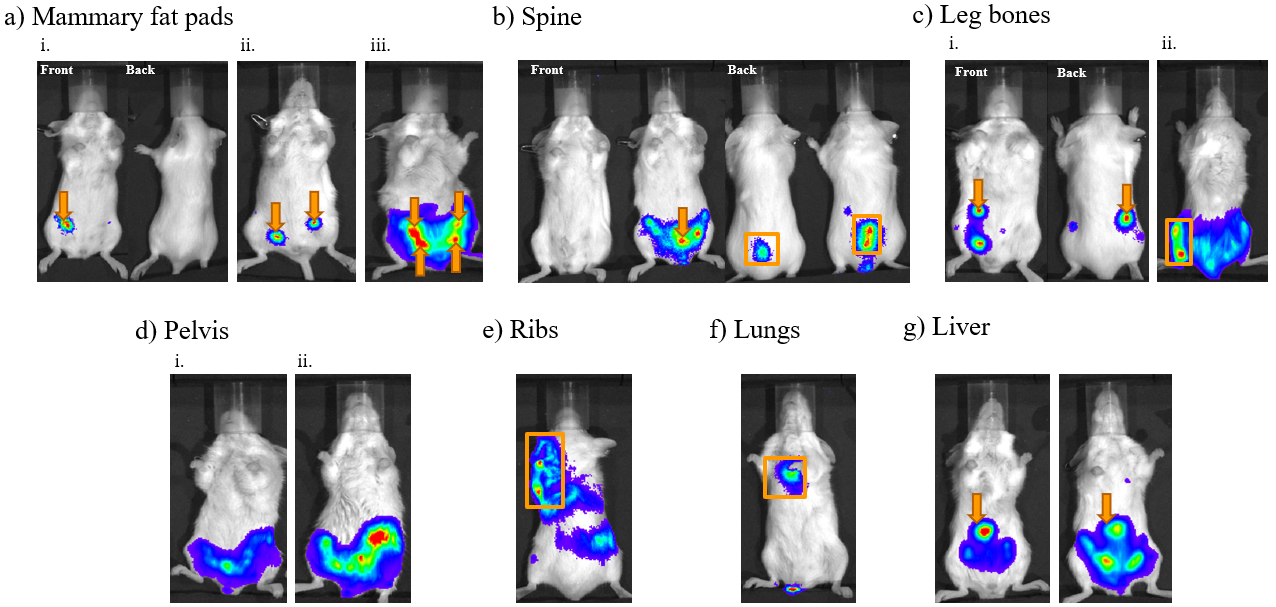


**Supplemental Figure 4.** Methodology for identifying metastasis location. a) i. Early mammary tissue (referred to as “fat pad”) metastases were identified using bioluminescent images of mice front side up. ii. Bioluminescent signals appeared circular and were positioned on abdominal mammary fat pads. iii. Distinct tumors in all four lower abdominal fat pads were identifiable even in later stages of tumor growth. b) Spine metastases were identified from imaging the back sides of mice. Signals from the spine appeared as elongated along the middle of the mouse (between the lower inguinal fat pads) and were exclusively found on the lower spine. c) i. Early signals indicating leg metastases were typically circular and seen on the lateral edge of the lower abdomen from both the front and back of the animal. ii. These signals began as circular foci around the ball of the femur before moving down the bone as the tumor developed. d) Pelvic metastases can be difficult to identify because the location is surrounded by sites of high metastasis, such as fat pads. The primary identifying mark of pelvic metastases were curved signals looping down and up towards the hip of the animal and may lack clear foci. e) Metastases to the ribs were rare and were strongest towards the edges of the chest. f) Metastases to the lungs are clearly visible on either side of the midline of the chest. These were characterized by a strong signal from the front of the mouse and little to no signal from the back of the animal. g) Signals which originated higher than the pelvis of the animal, but lower than the diaphragm, were considered to have metastasized to the liver. Liver signals were observed on the front of the abdomen as circular and slightly off-center towards the right side of the animal. Orange Arrow/Box: Indicates labeled signal. Images marked with “front” or “back” denote the same mouse, and images separated by a space indicate different mice.

**Supplemental Video 1.** Full demonstration of caudal artery injection.
